# A prospective observational study comparing a non-operator dependent automatic PWV analyser to pulse pressure, in assessing arterial stiffness in hemodialysis

**DOI:** 10.1186/s12882-015-0058-9

**Published:** 2015-04-23

**Authors:** Igor Salvadé, Sibylle Schätti-Stählin, Eleonora Violetti, Carlo Schönholzer, Claudio Cereghetti, Hugo Zwahlen, Lorenzo Berwert, Michel Burnier, Luca Gabutti

**Affiliations:** Division of Nephrology, Ospedale la Carità, Via Ospedale 1, 6600 Locarno, Switzerland; Division of Nephrology, Ospedale Civico, Lugano, Switzerland; Division of Nephrology, Ospedale Beata Vergine, Mendrisio, Switzerland; Division of Nephrology, Ospedale San Giovanni, Bellinzona, Switzerland; Division of Nephrology, University Hospital of Lausanne, Lausanne, Switzerland

**Keywords:** Arterial stiffness, Hemodialysis, Mobil-o-graph, Pulse pressure, Pulse wave analysis, Pulse wave velocity

## Abstract

**Background:**

Chronic kidney disease (CKD) accelerates vascular stiffening related to age. Arterial stiffness may be evaluated measuring the carotid-femoral pulse wave velocity (PWV) or more simply, as recommended by KDOQI, monitoring pulse pressure (PP). Both correlate to survival and incidence of cardiovascular disease. PWV can also be estimated on the brachial artery using a Mobil-O-Graph; a non-operator dependent automatic device. The aim was to analyse whether, in a dialysis population, PWV obtained by Mobil-O-Graph (MogPWV) is more sensitive for vascular aging than PP.

**Methods:**

A cohort of 143 patients from 4 dialysis units has been followed measuring MogPWV and PP every 3 to 6 months and compared to a control group with the same risk factors but an eGFR > 30 ml/min.

**Results:**

MogPWV contrarily to PP did discriminate the dialysis population from the control group. The mean difference translated in age between the two populations was 8.4 years. The increase in MogPWV, as a function of age, was more rapid in the dialysis group. 13.3% of the dialysis patients but only 3.0% of the control group were outliers for MogPWV. The mortality rate (16 out of 143) was similar in outliers and inliers (7.4 and 8.0%/year). Stratifying patients according to MogPWV, a significant difference in survival was seen. A high parathormone (PTH) and to be dialysed for a hypertensive nephropathy were associated to a higher baseline MogPWV.

**Conclusions:**

Assessing PWV on the brachial artery using a Mobil-O-Graph is a valid and simple alternative, which, in the dialysis population, is more sensitive for vascular aging than PP. As demonstrated in previous studies PWV correlates to mortality. Among specific CKD risk factors only PTH is associated with a higher baseline PWV.

**Trial registration:**

ClinicalTrials.gov Identifier: NCT02327962.

## Background

Chronic hemodialysis patients should have arterial stiffness evaluated monthly using Pulse Pressure as suggested by KDOQI guidelines [1]. This recommendation pursues a dual goal since it outlines the importance of monitoring vascular stiffness in hemodialysis patients [2-4] and, at the same time, emphasizes the fact that Pulse Pressure provides valuable information on tissue perfusion characteristics [5]. Arterial stiffening in dialysis patients is the result of aging and of non-specific and End Stage Renal Disease (ESRD) related risk factors, such as medial calcification, volume overload, uraemia-related endothelial dysfunction, increased extracellular matrix and intimal fibroelastic thickening [6]. Arterial stiffness of the aorta and its major branches can be evaluated by measuring Pulse Wave Velocity (PWV) – e.g. carotid-femoral Pulse Wave Velocity (“gold standard”) [7] – or, alternatively, it can be estimated by Pulse Wave Analysis (PWA) at a peripheral site, usually the brachial artery [8-11]. In the first case, a doppler ultrasound detector is used together with a software tool for data analysis. The second approach requires the use of a sphygmomanometer, such as the Mobil-O-Graph, capable of analysing the pulse wave morphology and of calculating PWV [9-13]. The first methodology is complex, operator-dependent and not routinely applicable, whereas the second one is potentially exploitable in clinical practice [9].

Coming back to arterial stiffness it is also important to mention that the increase in PWV related to the above-mentioned risk factors accelerates with age [7] and that both, PWV and PP, correlate to mortality in the dialysis population [14-16]. For each PWV increase of 1 m/s Blacher et al. found an all-cause mortality-adjusted OR of 1.39 (95% CI, 1.19 to 1.62) [15] while for each 10 mmHg increase in PP, Tozawa et al. found an increase in all-cause mortality relative risk of 8% [16].

Risk factors such as age, hypertension, previous history of heart diseases and diabetes influence the evolution of Pulse Wave Velocity before dialysis initiation whereas their impact during the course of dialysis has not yet been demonstrated [17]. In this regard, a study published in 2013 by Utescu et al. indicated that the only risk factor significantly associated with PWV progression was the level of an advanced glycation end-product known as pentosidine [17]. The results of this study confirmed that specific uraemia-related risk factors can be identified and possibly quantified.

In the above-mentioned study, the rate of PWV progression (+0.84 m/s per year) was surprisingly high, especially when projected over time as a function of the average life span of ESRD patients on dialysis. Another critical data point outlined in the study was the discrepancy in the annual rate of change in carotid-femoral compared to carotid-radial Pulse Wave Velocity, which was +0.84 m/s per year and −0.66 m/s per year, respectively. The authors of the study postulate that this discrepancy may be due to anatomical differences between central (elastic) and peripheral (muscular) arteries and that the latter could deploy an adaptive response to central aortic stiffening. Although interesting, these data raise some concerns about the promising possibility of using the brachial artery as a site for PWV estimation, even if based on a non-operator dependent method.

Furthermore, another limitation identified in the literature currently available on prospective longitudinal studies analysing the PWV behaviour on dialysis patients, is the lack of a control group made up of patients with similar characteristics and co-morbidities but without, or with a less severe, kidney disease [2,15,17].

In the light of this, we decided to test a Mobil-O-Graph, a simple device estimating PWV (MogPWV) through a modified sphygmomanometer on the brachial artery and to analyse the baseline and follow-up MogPWV values in a cohort of dialysis patients and in a control group with the same risk factors but an eGFR > 30 ml/min.

The aim of the study was answering the following 4 questions, which also reflect both the primary and the secondary endpoints of the trial: 1. Is PWV estimated by Mobil-O-Graph on the brachial artery more sensitive for vascular aging and discriminates better the dialysis population from the control group than pulse pressure? (primary endpoint); 2. Is MogPWV progression during dialysis faster compared to the rate calculated as a function of age on the basis of the data obtained at the inclusion day? (secondary endpoint); 3. Are there specific risk factors that correlate to baseline MogPWV? (secondary endpoint); 4. Does mortality correlate to MogPWV? (secondary endpoint).

## Methods

According to the above cited aims, a multi-centre cohort study was designed.

Hemodialysis patients were recruited from 4 dialysis units located in the Italian-speaking part of Switzerland (Ospedale la Carità, Locarno; Ospedale San Giovanni, Bellinzona; Ospedale Civico, Lugano; Ospedale Beata Vergine, Mendrisio), starting from January 2011. The study was designed to last 30 months. Subjects of the control group were recruited from patients hospitalized at Ospedale la Carità, Locarno, for elective minor surgery and waiting for transfer or discharge.

Inclusion criteria for the hemodialysis group: age 18 and older; ability to understand the information presented and to sign the informed consent; chronic hemodialysis for at least 8 weeks. Exclusion criteria for the hemodialysis group: mental illness; inability to understand the information presented and to sign the informed consent; acute disease requiring hospitalization at the time of patient enrolment; evidence of stenosis of the subclavian artery of the arm without shunt or of the non-shunt arm chosen to be used for the Mobil-O-Graph measurements; atrial fibrillation.

Inclusion criteria for the control group: age 18 and older; ability to understand the information presented and to sign the informed consent. Exclusion criteria for the control group: mental illness; inability to understand the information presented and to sign the informed consent; evidence of stenosis of both subclavian arteries; atrial fibrillation; eGFR(EPI) < 30 ml/min (CKD stage 4 and 5).

Non-invasive hemodynamic and MogPWV measurements were performed the day of inclusion in the study and repeated every 3 months the first year and then every 6 months (the non-shunt arm or the right arm being chosen for the haemodynamic measurements), together with a comprehensive screening of general (hypertension, diabetes, dyslipidaemia, smoking habit, history of cardiovascular disease) and specific cardiovascular risk factors for CKD patients (serum calcium, phosphate and PTH). At the inclusion, anamnestic data about the cause of kidney disease (biopsy proven or assumed as by history and clinical data), data of dialysis initiation, dialysis modality and prescriptions (including ultrafiltration volume), dialysis quality (using the kt/V; Daugirdas), medications including phosphate binders, vitamin D and analogues, calcium agonists and antihypertensive and routine laboratory tests (including hemoglobin, bicarbonate and lipid profile) were also collected. At the follow-up visits file information was upgraded and changes protocolled.

The Mobil-O-Graph (I.E.M. GmbH, Stolberg, Germany), an electronic blood pressure monitoring system equipped with an arm cuff and a software tool for Pulse Wave Analysis (calculating PWV, augmentation index and the reflection coefficient) was chosen for non-invasive hemodynamic and PWV monitoring. The reliability of the Mobil-O-Graph in estimating the PWV by PWA (MogPWV) was demonstrated in previous studies [10-12,18-22]. Hemodynamic parameters (peripheral and central blood pressure, cardiac output, peripheral vascular resistance) and MogPWV were recorded for each subject 10 times randomly distributed during the course of dialysis (or during the day before hospital discharge for the patients of the control group) and mean values were obtained. Eight Mobil-O-Graph were employed for the study. Calibration was guaranteed by the supplying company.

Statistical analysis was performed using the R software tool [23]. Starting from a multivariate model of MogPWV as a function of all measured variables, using a "backward-elimination", it was established that the essential variables of the model were age and systolic blood pressure according to the formula: *E*[*MogPWV*] = *α* + *β*_1_ * *age* + *β*_2_ * *PA*_*syst*_. The MogPWV values were then normalized by eliminating the effect of systolic blood pressure, on the basis of the above cited equation, independently obtained from the results of the two groups. Aware of the fact that systolic blood pressure is a parameter already used in the algorithm to calculate the MogPWV by PWA, normalisation was applied considering the strong intra-individual influence of systolic blood pressure on MogPWV and the extreme variability of blood pressure during dialysis. MogPWV outliers were defined as individuals with, Bonferroni corrected studentized residuals *P* values (of the measured PWV) of less than 0.05.

To test the normality of the residuals the Shapiro’s test and the QQ-plot were used while the homoscedasticity was proven thanks to the Levene (for the homogeneity of the variances) and the Breusch-Pagan (for the residuals of the models) tests [24]. To verify the absence of serial correlation of the residuals and their independence, the Durbin-Watson test was used [25]. Hemodynamic parameters and laboratory results were compared using a t-test and a Mann–Whitney U test. Contingency tables were analysed using the chi-square while the significance of the odds-ratios was explored using the two-sided Fisher's Exact Test [25].

In all cases, a *P* value ≤ 0.05 was considered statistically significant.

The protocol of the study was approved by the local ethics committee (Ethical Committee of the Canton Ticino, CE 2550). Informed consent was obtained from all patients prior to enrolment.

## Results

### Characteristics of the population

143 patients were included in the dialysis group and 100 in the control group (see Table 1 for details and comparisons). Gender distribution and the percentage of patients treated with RAAS-inhibitors and alpha-blockers were the only significant difference between groups. The presumed aetiology of ESRD in the dialysis group, in descending order, was: hypertensive nephropathy 30%, diabetic nephropathy 25%, glomerulonephritis 15%, polycystic kidney disease 12%, other identified causes 8%, obstructive or post-pyelonephritic nephropathy 6%, interstitial nephropathy 5%, unknown 3% (the aetiology of renal disease was biopsy proven in 32% of the patients only).

**Table 1:** **Characteristics of the study population**

	**Dialysis Group (N = 143)**	**Control Group (N = 100)**	***P*** **-value**
**Age** *yr*	71.3 ± 11.9	72.0 ± 8.9	ns
**Male sex** *no. (%)*	77 (53.8)	71 (71.0)	<0.01
**Coexisting conditions** *no. (%)*			
**Smoker**	47 (32.9)	45 (45.0)	ns
**pack years**	44.4 ± 19.6	57.6 ± 23.1	ns
**Hypertension**	130 (90.9)	86 (86.0)	ns
**Diabetes**	54 (37.8)	32 (32.0)	ns
**HbA1c %**	6.9 ± 1.6	6.6 ± 1.8	ns
**Dyslipidaemia**	86 (60.1)	52 (52.0)	ns
**Cholesterol total** *mmol/L*	4.5 ± 1.2	4.6 ± 1.5	ns
**LDL cholesterol** *mmol/L*	2.7 ± 1.0	3.1 ± 1.3	ns
**CVD**	83 (58.0)	53 (53.0)	ns
**eGFR (EPI)** *ml/min/1.73 m2*	<15	67.2 ± 23.9	-
**Proteinuria** *mg/mmol*	No data	23 ± 47	-
**Anti-hypertensive therapy** *no. (%)*			
**RAAS-inhibitors**	64 (44.8)	64 (64.0)	<0.01
**Calcium-antagonists**	48 (33.6)	30 (30.0)	ns
**Beta-blockers**	61 (42.7)	35 (35.0)	ns
**Alpha-blockers**	29 (20.3)	4 (4.0)	<0.01
**Diuretics**	72 (50.3)	43 (43.0)	ns
**Follow-up** *months*	16.4 ± 7.5	0	-
**Duration of dialysis** *months*	40.3 ± 35.2	-	-
**Kt/V**	1.47 ± 0.33	-	-
**Haemodynamics measures** *mean* ± *SD*			
**Systolic Pressure (peripheral)** *mmHg*	126.2 ± 22.9	128.7 ± 17.5	ns
**Diastolic Pressure (peripheral)** *mmHg*	74.8 ± 14.0	78.4 ± 12.5	<0.05
**Systolic Pressure (central)** *mmHg*	114.8 ± 21.1	117.8 ± 16.6	ns
**Diastolic Pressure (central)** *mmHg*	76.5 ± 14.1	79.9 ± 12.6	0.05
**Peripheral resistances** s*mmHg/ml	1.4 ± 0.3	1.4 ± 0.2	ns
**Cardiac output** *l/min*	3.8 ± 0.6	3.9 ± 0.6	ns
**Pulse Pressure** *mmHg*	51.3 ± 15.1	50.3 ± 12.0	ns
**Diabetics**	53.5 ± 10.5	54.6 ± 12.9	ns
**non-Diabetics**	50.0 ± 17.2	48.3 ± 11.2	ns
**CVD**	50.8 ± 14.4	49.5 ± 12.1	ns
**non-CVD**	52.0 ± 16.2	51.1 ± 12.0	ns
**Alx@75** *%*	25.1 ± 10.6	29.6 ± 10.0	<0.01
**Refl. Coef.** *%*	63.1 ± 7.5	64.8 ± 6.3	ns (0.06)
**MobPWV** adjusted *m/s*	7.6 ± 2.1	6.4 ± 1.5	<0.01
**Diabetics**	7.9 ± 1.9	6.5 ± 1.5	<0.01
**non-Diabetics**	7.5 ± 2.2	6.4 ± 1.6	<0.01
**CVD**	7.8 ± 2.2	6.7 ± 1.4	<0.01
**non- CVD**	7.4 ± 2.0	6.1 ± 1.6	<0.01
**MobPWV** unadjusted *m/s*	10.6 ± 2.2	10.5 ± 1.7	ns
**Diabetics**	10.9 ± 2.0	10.7 ± 1.7	ns
**non-Diabetics**	10.5 ± 2.3	10.4 ± 1.7	ns
**CVD**	10.7 ± 2.3	10.8 ± 1.7	ns
**non-CVD**	10.5 ± 2.1	10.2 ± 1.6	ns
**Labor results** *mean* ± *SD*			
**Ionized calcium** *mmol/l*	1.15 ± 0.09	*No data*	
**Diabetics**	1.14 ± 0.08		
**non-Diabetics**	1.16 ± 0.09		
**CVD**	1.15 ± 0.09		
**non-CVD**	1.15 ± 0.09		
**PTH** *pmol/l*	28.9 ± 21.0	*No data*	
**Diabetics**	26.1 ± 20.8		
**non-Diabetics**	30.6 ± 21.0		
**CVD**	28.8 ± 22.6		
**non-CVD**	29.0 ± 18.7		
**Phosphate** *mmol/L*	1.59 ± 0.44		
**Bicarbonate** *mmol/L*	18.7 ± 8.1		

MobPWV = PWV obtained by Mobil-O-Graph (adjusted by systolic blood pressure and unadjusted); Alx@75 = augmentation index corrected at 75 beats per minute; Refl. Coef. = reflection coefficient; CVD = cardiovascular disease (coronary heart disease and/or cerebrovascular disease and/or peripheral arterial disease); CHF = congestive heart failure; BMI = body-mass Index; RAAS = renin-angiotensin-aldosterone-system; DBW = dry body weight; ns = non-significant.

### Intra-patient MogPWV value dispersion

Reproducibility of results was confirmed by the small dispersion of the normalized intra-individual MogPWV values (0.388 ± 0.264 and 0.450 ± 0.304 m/s for the dialysis and control group respectively).

### Relationship between MogPWV and Systolic Blood Pressure

Systolic blood pressure significantly influenced MogPWV in both groups: in the control group a systolic increase of 10 mmHg generates a MogPWV increase of 0.319 ± 0.014 m/s. The dialysis group featured a lower increase, i.e. 0.237 ± 0.025 m/s (*P* < 0.01).

### *Discrimination sensitivity* (primary endpoint)

**Figure 1:**
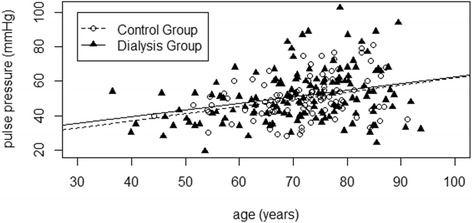
Pulse Pressure and age. Pulse Pressure progression as a function of age in both groups (*P* between groups n.s.).

**Figure 2:**
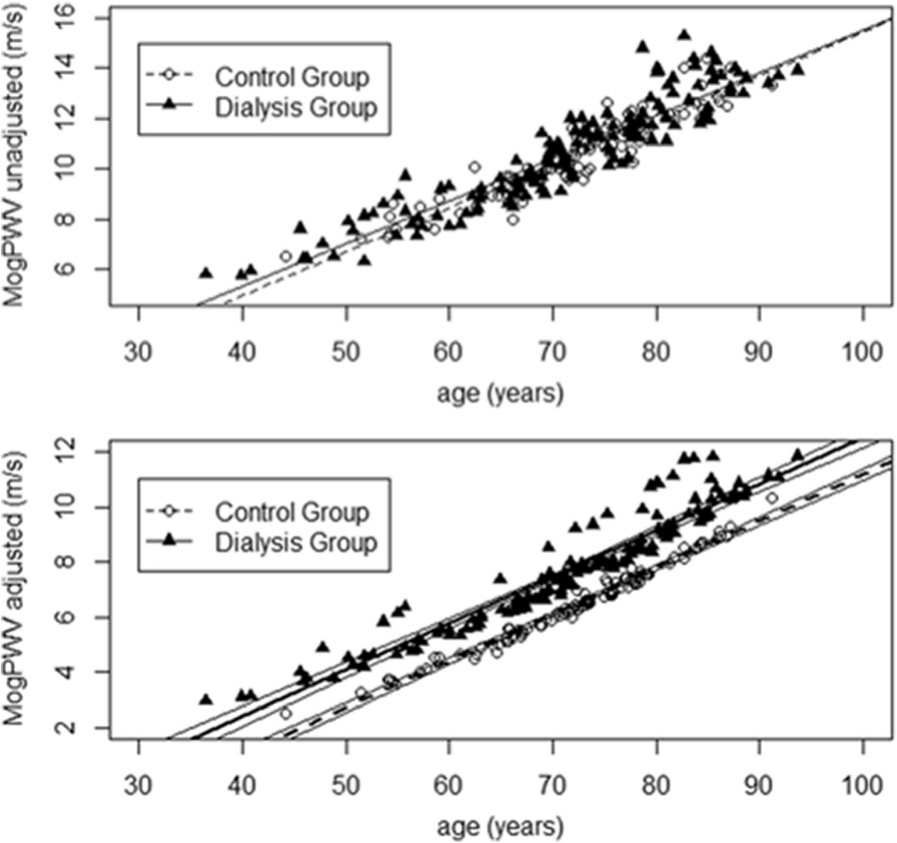
Pulse Wave Velocity and age. MogPWV progression as a function of age in both groups. Native values above (*P* between groups n.s.); after adjusting by systolic blood pressure below (*P* between groups <0.001).

Unlike Pulse Pressure (*P* between groups *n.s.* on a PP against age distribution), MogPWV significantly discriminates the dialysis population from the control group (*P* <0.001) (Figures 1 and 2). 13.3 % (19 out of 143) of the dialysis patients were outliers for MogPWV vs. 3.0% (3 out of 100) in the control group (*P* 0.001); signifying that 13.3% of the dialysis patients, compared to the rest of the group, had a statistically significant unexpected elevated MogPWV value.

### MogPWV and aging

The mean horizontal difference between the two MogPWV against age distributions (depicting the behaviour of the dialysis and control group) was 8.4 years (95% CI: 3.8-12.9) meaning that the arteries of our dialysis population were, on average, 8.4 years older than controls. These findings suggest that the difference is exclusively related to the extent of renal function impairment, since other risk factors were comparable across the two groups. The regression line for the dialysis group, divided into 4 quartiles of age, was slightly but significantly steeper than in the control group (0.230 ± 0.008 vs. 0.199 ± 0.008 m/s per year; *P* 0.05); while the slope increases with age in both groups.

### *Mortality rate* (secondary endpoint)

**Figure 3:**
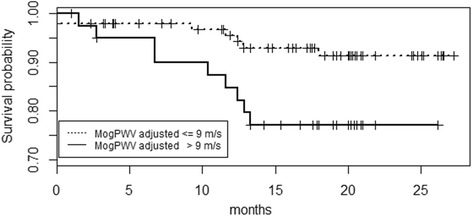
Pulse Wave Velocity and survival. Survival as a function of MogPWV. Kaplan-Meier curve depicting the probability of survival in two subgroups of MogPWV (*P* 0.02; Relative risk 2.96).

16 out of 143 dialysis patients died during the follow-up. The aetiology was CVD in 9, oncological diseases in 3, infection in 3 and severe post-operative bleeding in 1. The mortality rate (11.2%) was similar in outliers and inliers (7.4 and 8.0 % per year). Patient stratification according to the MogPWV showed a significant difference in survival (Figure 3).

### *Risk factors for higher baseline MogPWV* (secondary endpoint)

Hypertensive nephropathy as a presumed cause for ESRD (the systolic blood pressure corrected MogPWV in hypertensive nephropathy, diabetic nephropathy, glomerulonephritis, polycystic kidney disease, obstructive or post-pyelonephritic nephropathy and interstitial nephropathy was respectively: 8.606 ± 1.904, 7.587 ± 1.836, 7.285 ± 2.120, 6.616 ± 2.045, 7.228 ± 2.434 m/s), higher PTH levels (inliers 27.4 ± 20.5; outliers 38.7 ± 21.8 pmol/L; *P* 0.03), lower serum calcium (inliers 1.16 ± 0.08; outliers 1.09 ± 0.11 mmol/L; *P* <0.001), lower serum phosphate (inliers 1.60 ± 0.46; outliers 1.48 ± 0.27 mmol/L; n.s.; P < 0.05 in the dichotomous analysis; Table 2 ) and lower dialysate calcium levels (*P* <0.001) correlate with higher baseline MogPWV. See Table 2 for details.

**Table 2:** **Analysis of the potential risk factors for a higher PWV value (for dichotomous variables); CVD = cardiovascular disease (coronary heart disease and/or cerebrovascular disease and/or peripheral arterial disease); ns = non-significant**

		**Inliers**	**Outliers**	**P-value**	**OR**	**CI 95%**
**Dialysate calcium**	Low *(1.25 mmol/L)*	49	19	< 0.001		
	High *(1.50 mmol/L)*	74	0			
**Diabetes**	Yes	51	3	ns (0.06)	3.63	1.03-13.45
	No	73	16			
**Smoking habit**	Yes	37	10	ns (0.08)	2.61	0.98-6.96
	No	87	9			
**CVD**	Yes	73	10	ns	1.29	0.49-3.39
	No	51	9			
**Hypertension**	Yes	113	17	ns	1.21	0.25-5.93
	No	11	2			
**Dyslipidaemia**	Yes	75	11	ns	1.11	0.42-2.96
	No	49	8			
**Serum Phosphate**	Low *(<1.79 mmol/L)*	91	18	<0.05	6.47	1.03-138.1
	High *(>1.78 mmol/L)*	33	1			

### *Progression of MogPWV in dialysis* (secondary endpoint)

**Figure 4:**
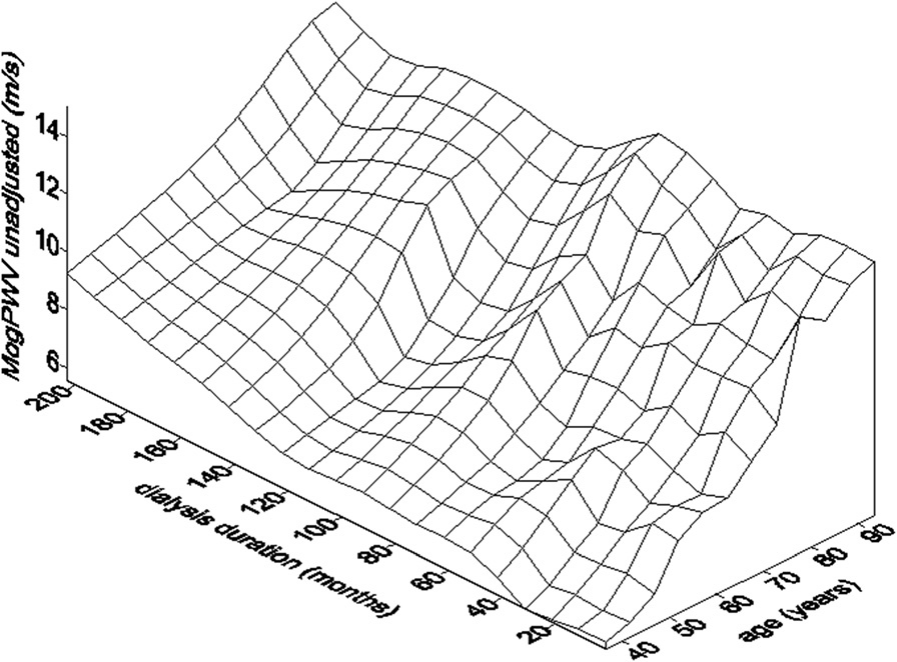
Pulse Wave Velocity and dialysis duration. Surface-plot showing the relationship among months on dialysis, age and MogPWV.

**Figure 5:**
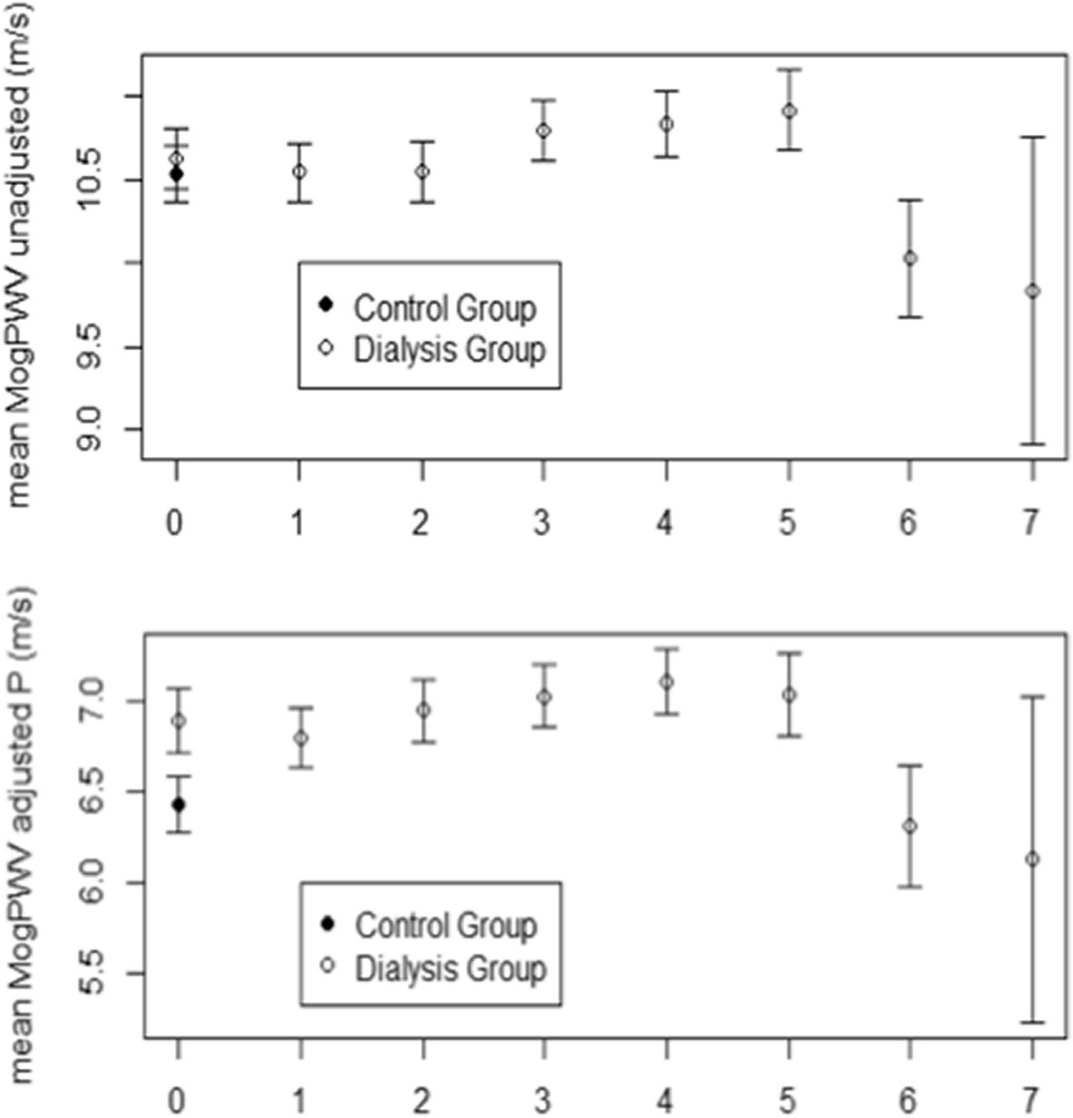
Pulse Wave Velocity at the inclusion and follow up visits. MogPWV are systolic blood pressure adjusted (above) and unadjusted (below) values respectively. 0: inclusion visits for subjects of the control and dialysis groups respectively; 1–7: follow-up visits for the dialysis group. Values are given in mean; SD is depicted.

**Figure 6:**
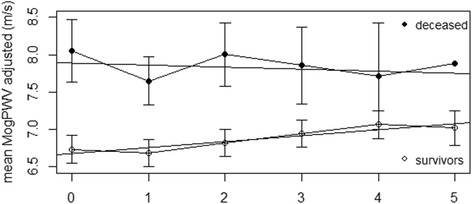
Comparison of MogPWV progression rate between survivors and deceased of the dialysis group. MogPWV are systolic blood pressure adjusted values. 1–5: follow-up visits. None of the deceased was evaluated at visit 6 and 7. Values are given in mean; SD is depicted.

The progression of MogPWV in dialysis did not significantly differ from the rate calculated, as a function of age, thanks to the regression model using the data of the inclusion day (0.165 ± 0.003 vs. 0.169 ± 0.002 m/s per year) (see Figure 4 for the surface plot and Table 3 and Figure 5 for the details of MogPWV evolution as a function of the follow-up visits). Comparing MogPWV progression rate between survivors and deceased, a significant difference was not demonstrated (see Figure 6).

**Table 3:** **Haemodynamics parameters at inclusion and follow-up**

	**Control Group**	**Dialysis Group**							
	**Enrollment visit**		**Follow-up visits**						
	**C 0**	**D 0**	**D 1**	**D 2**	**D 3**	**D 4**	**D 5**	**D 6**	**D 7**
Number of subjects	100	143	130	120	112	99	66	17	6
**Age** *years* (mean ± SD)	72.0 ± 8.9	71.1 ± 11.9	72.1 ± 11.4	73.3 ± 10.7	73.6 ± 10.4	74.0 ± 10.2	73.9 ± 10.1	70.1 ± 8.0	68.7 ± 12.5
Median	73.1	72.0	73.2	73.8	74.0	74.2	73.7	70.4	66.7
1st Qu.	66.2	65.4	66.3	67.5	68.2	68.6	68.0	64.7	59.6
3rd Qu.	77.6	80.1	80.5	80.9	81.1	81.4	81.1	74.4	76.4
**MobPWV unadjusted** *m/s* (mean ± SD)	10.5 ± 1.7	10.6 ± 2.2	10.5 ± 2.0	10.5 ± 2.0	10.8 ± 1.9	10.8 ± 1.9	10.9 ± 2.0	10.0 ± 1.4	9.8± 2.2
Median	10.65	10.80	10.60	10.50	10.80	11.00	11.00	10.10	9.40
1st Qu.	9.40	9.08	9.30	9.10	9.70	9.65	9.38	9.25	8.05
3rd Qu.	11.73	12.00	12.00	11.83	12.10	12.20	11.90	11.05	10.90
**MobPWV adjusted** *m/s* (mean ± SD)	6.4±	6.9 ± 2.1	6.8±	6.9±	7.0±	7.1±	7.0±	6.3±	6.1±
Median	6.48	6.85	6.83	6.91	7.13	7.12	6.87	6.29	5.70
1st Qu.	5.37	5.53	5.52	5.70	5.79	6.07	5.81	5.39	4.28
3rd Qu.	7.37	8.39	8.07	8.31	8.37	8.42	8.14	7.07	7.71
**Pulse Pressure** *mmHg* (mean ± SD)	50.3 ± 12.0	51.4 ± 15.1	51.7 ± 15.5	49.2 ± 13.5	53.2 ± 13.9	52.2 ± 14.0	55.4 ± 12.9	50.5 ± 9.5	50.4 ± 7.0
Median	49.0	50.0	50.6	46.6	52.2	50.4	53.4	51.3	48.6
1st Qu.	42.8	41.0	40.5	39.3	44.3	41.8	46.2	43.1	44.7
3rd Qu.	55.5	59.9	61.3	56.2	60.7	59.7	64.6	56.8	56.5
**Systolic BP** peripheral *mmHg* (mean ± SD)	128.7 ± 17.5	126.2 ± 22.9	126.3 ± 22.1	121.4 ± 20.4	126.7 ± 21.3	125.6 ± 22.3	130.6 ± 19.5	125.2 ± 16.3	124.7 ± 12.3
Median	127.5	124.0	124.5	118.0	124.0	127.0	129.5	126.0	123.5
1st Qu.	116.0	110.5	111.8	108.0	114.0	110.8	117.0	111.0	114.3
3rd Qu.	139.3	142.0	140.3	133.0	139.0	140.0	143.0	138.0	135.8
**Diastolic BP** peripheral *mmHg* (mean ± SD)	78.4 ± 12.5	74.8 ± 14.0	74.7 ± 12.9	72.2 ± 12.0	73.4 ± 12.8	73.5 ± 13.0	75.3 ± 12.0	74.8 ± 12.1	74.3 ± 10.7
Median	80.0	72.0	74.5	70.0	73.0	73.0	76.0	74.0	71.5
1st Qu.	70.0	65.0	66.0	64.0	65.0	65.0	65.0	67.0	69.3
3rd Qu.	86.0	84.0	82.3	79.0	81.8	82.3	82.5	82.0	77.5

C 0 and D 0: inclusion visits for subjects of the control and dialysis groups respectively; D 1–7 follow-up visits for the dialysis group. The patients were included progressively and for this reason, even if the drop out was only due to deaths, the number of patients decreases from visit 1 to visit 7.

### MogPWV and ultrafiltration rate

**Figure 7:**
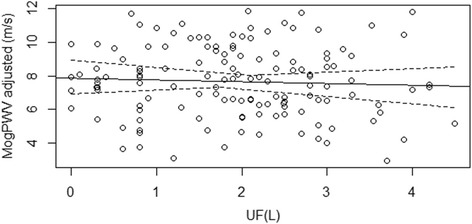
Relationship between MogPWV and Ultrafiltration. A correlation was not demonstrated.

The ultrafiltration rate did not correlate with MogPWV (see Figure 7).

## Discussion

The main goal of the study was to determine whether Pulse Wave Velocity estimated by Pulse Wave Analysis on the brachial artery through a Mobil-O-Graph, a modified sphygmomanometer, is more sensitive for vascular aging and arterial stiffness and therefore better than Pulse Pressure, the KDOQI guidelines recommended parameter for dialysis patient monitoring, in discriminating a cohort of dialysis patients from a control group with the same co-morbidities but with a significantly better renal function (stage 3 or less). The results furthermore confirm that PWV estimated by Mobil-O-Graph (MogPWV) is able to intercept a discriminating vascular aging parameter between dialysis patients and control group, which is otherwise undetectable via PP measurement. The 8.4 years difference between the two MogPWV distributions (95% CI 3.8-12.9 years) suggests that the arteries of our dialysis patients were, on average, 8.4 years older compared to a control group with a similar co-morbidity profile but a less compromised renal function.

Even if the difference in the antihypertensive medication profile between the two groups (more RAAS inhibitors in the control group and more alpha-blockers in the dialysis group), could have increased the magnitude of the difference in vascular aging [26,27], the potential source of bias should have been blunted by the adjustment for systolic blood pressure applied to MogPWV.

Extrapolating the results of the two curves obtained by the exponential model and expressing the distribution of the MogPWV as a function of age in the two groups, the theoretical convergence point was found to be located many decades before the start of dialysis. Taking into account the limits of such an analysis approach and the potential sources of bias, the result suggests that the acceleration of PWV worsening begins long before the onset of ESRD.

The results obtained, substantiate the efficacy of using such a simple non-operator dependent device to measure vascular stiffness in haemodialysis patients in clinical wards.

However, the evidence gathered from this trial, as well as previous ones, does not make it possible to ascertain the extent to which MogPWV estimated on the brachial artery, is representative of the actual carotid-femoral PWV for this particular patient setting.

Even if a correlation between MogPWV and ultrafiltration rate during dialysis, in the present study, was not demonstrated; we must also bear in mind that PWV is subject to fluctuations during dialysis due to changes in blood pressure, solute concentrations and circulating volume [28-31]. Even before the start of the dialysis session and during the interdialytic interval, the results obtained might be perturbed by the variability of blood pressure and by volume overload. Furthermore, in the dialysis setting it is obviously not possible to perfectly conform to the recommendation 11 (recommendations 3 and 9 are not pertinent to the use of a Mobil-O-Graph) of the current guidelines to assess PWV, stating to avoid measurements in unstable clinical situations [32].

Although our results were gathered during dialysis with many potential sources of bias, they provide interesting and intra-patients reproducible information on vascular aging that could be of relevance for individual patients. Therefore, despite the above-mentioned constraints, the results of this trial give the basis for the concrete selection of patients potentially at high-risk (a subgroup of outliers, accounting for 13.3% of our dialysis population, who showed MogPWV levels above the 95% CI), who could benefit from a customized management in the attempt to slow down vascular aging and related factors like vascular calcification; and this even if we were unable to demonstrate, probably because of the relatively small size of the population, a relationship between the outlier status and specific known risk factors (except for elevated PTH levels).

In a previous longitudinal study in dialysis, Utescu et al. were in turn unable to demonstrate the impact of classical risk factors on PWV progression; this eventually being also related to the limited caseload or to the fact that the largest amount of pathological changes in the vascular wall could have been accumulated years before the beginning of dialysis.

Other risk factors identified during the trial, such as serum ionized calcium under the lower limit of the normal distribution, serum phosphate ≤ 1.78 mmol/L, and physiological vs. supra-physiological dialysate calcium, were unexpected and may have been the result of the treatment strategy deployed during dialysis (e.g. physiological dialysate calcium is preferred in the absence of intradialytic hypotension or other specific indications for a positive calcium balance). Moreover, as suggested above, the risk factors were detected during the trial period and did not account for past or pre-dialysis patient management strategies that might explain the MogPWV outlier status. Emphasizing this hypothesis, evidence of hypertensive nephropathy, although not biopsy proven in most of the cases, as the aetiology of End Stage Renal Disease , correlated to higher MogPWV, as expected.

Since this trial shows no evidence of faster MogPWV progression during dialysis vs. pre-dialysis cumulative data (+0.165 vs. +0.169 m/s/year), we can assume, as mentioned above, that the difference vs. controls progressively built up over the years preceding dialysis initiation, most likely due to the severity of kidney dysfunction being a specific risk factor, and, possibly, to patient management strategies.

Moreover, the steeper curve depicting MogPWV as a function of Blood Pressure in the control group confirms that the vascular behaviour and the relationship PWV-Blood Pressure between groups is different. A lesser PWV reversibility decreasing systolic blood pressure in the dialysis group could be postulated.

Similarly to previous trials [15,33-35], mortality rates were higher among patients with elevated MogPWV. However age plays a fundamental role in MogPWV progression and, eventually due to the small number of patients who died during the trial period (16 out of 143; 11%), after adjusting for age, the difference in mortality between MogPWV subgroups was no longer statistically significant.

## Conclusions

Estimating PWV on the brachial artery using a Mobil-O-Graph is a valid and simple alternative to direct measurement, is more sensitive for vascular aging and discriminates better the dialysis population, identifying at the same time the outliers, than Pulse Pressure. The importance of early prevention of vascular stiffening is once again outlined by the facts that the arteries of our dialysis patients were, on average, 8.4 years older compared to a similar population but with a significant better renal function and that the acceleration of PWV progression has been found to begin well before dialysis initiation. As demonstrated in previous studies, MogPWV correlates to mortality. Among specific CKD risk factors, only PTH correlates to higher baseline MogPWV. Additional studies may contribute to further characterize risk factors, susceptible to being influenced, associated with MogPWV progression during dialysis.

## Abbreviations

CKD: Chronic kidney disease
ESRD: End stage renal disease
KDOQI: Kidney disease outcomes quality initiative
PWV: Pulse wave velocity
PWA: Pulse Wave analysis
PP: Pulse pressure
MogPWV: Pulse wave velocity obtained by mobil-o-graph
Alx@75: Augmentation index corrected at 75 beats per minute
Refl. Coef.: Reflection coefficient
CVD: Cardiovascular disease
CHF: Congestive heart failure
PTH: Parathormone
BMI: Body-mass index
DBW: Dry body weight
RAAS: Renin-Angiotensin-Aldosterone-System
